# Microbiota manipulation through the secretion of effector proteins is fundamental to the wealth of lifestyles in the fungal kingdom

**DOI:** 10.1093/femsre/fuac022

**Published:** 2022-05-23

**Authors:** Nick C Snelders, Hanna Rovenich, Bart P H J Thomma

**Affiliations:** Institute for Plant Sciences, University of Cologne, D-50674 Cologne, Germany; Theoretical Biology & Bioinformatics Group, Department of Biology, Utrecht University, 3584 CH Utrecht, The Netherlands; Institute for Plant Sciences, University of Cologne, D-50674 Cologne, Germany; Institute for Plant Sciences, University of Cologne, D-50674 Cologne, Germany; Cluster of Excellence on Plant Sciences, Institute for Plant Sciences, University of Cologne, D-50674 Cologne, Germany

**Keywords:** microbiota, dysbiosis, effector, fungus, ecology, interactions

## Abstract

Fungi are well-known decomposers of organic matter that thrive in virtually any environment on Earth where they encounter wealths of other microbes. Some fungi evolved symbiotic lifestyles, including pathogens and mutualists, that have mostly been studied in binary interactions with their hosts. However, we now appreciate that such interactions are greatly influenced by the ecological context in which they take place. While establishing their symbioses, fungi not only interact with their hosts but also with the host-associated microbiota. Thus, they target the host and its associated microbiota as a single holobiont. Recent studies have shown that fungal pathogens manipulate the host microbiota by means of secreted effector proteins with selective antimicrobial activity to stimulate disease development. In this review, we discuss the ecological contexts in which such effector-mediated microbiota manipulation is relevant for the fungal lifestyle and argue that this is not only relevant for pathogens of plants and animals but also beneficial in virtually any niche where fungi occur. Moreover, we reason that effector-mediated microbiota manipulation likely evolved already in fungal ancestors that encountered microbial competition long before symbiosis with land plants and mammalian animals evolved. Thus, we claim that effector-mediated microbiota manipulation is fundamental to fungal biology.

## Teaser

Recent findings show that fungal pathogens secrete effector molecules to manipulate the host microbiota to stimulate disease development; here, we argue that fungal effector-mediated microbiota manipulation occurs beyond plant pathogens, is likely widespread in the fungal kingdom and occurs in many contexts.

## INTRODUCTION

Complex multicellular organisms, including humans, animals and plants, associate with countless microbes that are collectively termed their microbiota, and which is an important determinant for their well-being (Bulgarelli *et al*. 2012, Huttenhower *et al*. 2012, Lundberg *et al*. 2012, Lloyd-Price *et al*. 2016). Microbiota members encompass a wealth of microbes that establish a broad spectrum of symbiotic relationships with their hosts, ranging from mutualistic through commensalistic to parasitic. Traditionally, symbiotic interactions between microbes and their hosts have predominantly been studied as one-on-one relationships between two partners, oftentimes focusing on pathogenic or mutualistic microbes.

Studies on the symbiotic interactions between plant-associated microbes and their hosts have similarly mainly been performed in binary contexts. From such studies, it has become evident that plant-associated microbes secrete small proteins, which are known as ‘effectors’, to promote host colonization (Giraldo and Valent 2013, Rovenich *et al*. 2014, Cook *et al*. 2015, Lo Presti *et al*. 2015). Initially, the term effector was exclusively used to describe pathogen-encoded small cysteine-rich *in planta*-secreted proteins that were proposed to be involved in the suppression of host immune responses. Ongoing research, however, revealed that effector proteins also exert other activities besides immune suppression that, for instance, involve self-protection from host-secreted antimicrobial compounds, scavenging of immune-stimulatory molecules or the manipulation of host physiology to liberate nutrients (van den Burg *et al*. 2006, de Jonge *et al*. 2010, Cox *et al*. 2017).

While effectors were initially considered as pathogen-specific proteins, it is increasingly recognized that effector proteins are not exclusively secreted by plant pathogens, but also by other types of symbionts, such as endophytes and mutualists, to establish their symbioses (Wawra *et al*. 2016, Nostadt *et al*. 2020, Zeng *et al*. 2020). This finding has challenged the initial dogma that nonpathogenic symbionts would not stimulate the host immune system and were therefore not targeted by host immune responses. However, increasing evidence has shown that mutualistic fungi, such as the mycorrhizal fungi *Laccaria bicolor* and *Glomus intraradices*, exploit effector proteins to target, for instance, jasmonate- or ethylene-dependent host immune responses (Kloppholz *et al*. 2011, Plett *et al*. 2020, Zeng *et al*. 2020). Perhaps more surprising is the finding that effectors are found in fungi that are not known to engage in symbiosis with plant hosts, such as saprotrophs. In fact, attempts to discriminate symbiotic fungi from their saprotrophic relatives based on the presence and size of effector repertoires have met little success (Lowe and Howlett 2012, Seidl *et al*. 2015). Even effectors that have been attributed roles in host colonization, such as chitin-binding LysM effectors and plant cell death-inducing NLP-type effectors, occur in the genomes of nonsymbiotic fungi (Gijzen and Nürnberger 2006, de Jonge and Thomma 2009, Kombrink and Thomma 2013, Seidl and van den Ackerveken 2019, Dubey *et al*. 2020, Suarez-Fernandez *et al*. 2021). Findings like these indicate that effectors are not (plant) symbiont-specific inventions to mediate (plant) host colonization, but should rather be seen as molecules that are secreted by fungi in order to manipulate their environment to their benefit. Arguably, for fungal symbionts, their hosts constitute important environments. This then implies that to colonize different hosts (or niches), a fungus requires effectors to support such changes in lifestyle. Indeed, fungi of the *Metarhizium* genus, which have the ability to switch between saprotrophic, plant endophytic and entomopathogenic lifestyles, employ suites of host- and organ-specific effectors to colonize those different niches (St Leger and Wang 2020).

**Figure 1.**
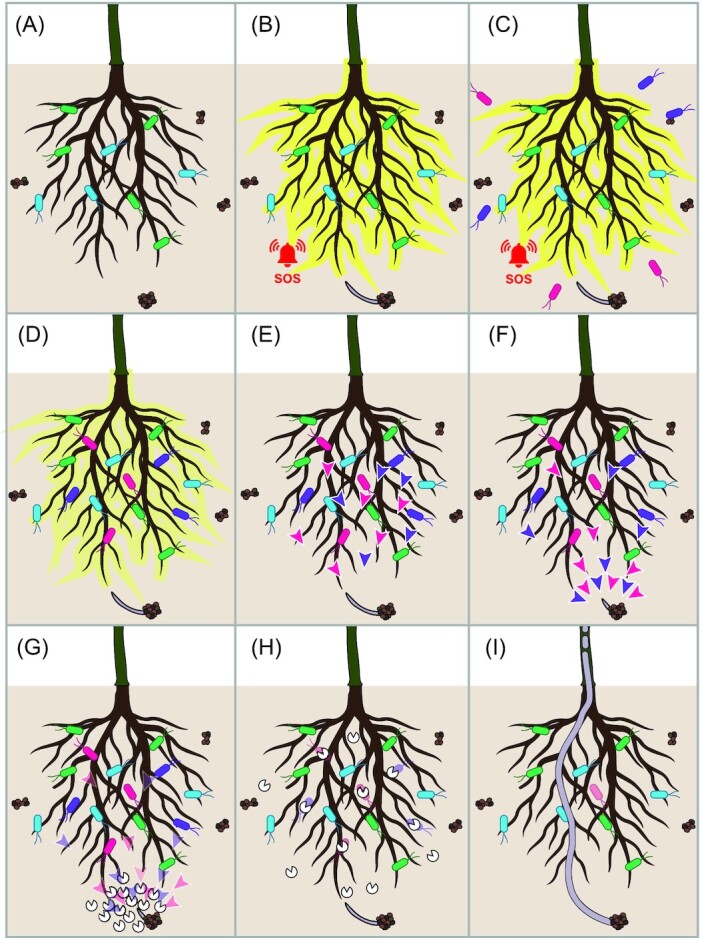
Fungal pathogens use effector proteins to counteract the recruitment of beneficial microbiota by their hosts. The well-being of complex multicellular organisms depends on the recruitment and maintenance of a balanced microbiota, which includes a multitude of bacteria and fungi with lifestyles ranging from mutualistic through commensalistic to parasitic. Host organisms survey their microbiota for potentially pathogenic invaders using various types of receptors that betray pathogen ingress and induce immune responses **(A, B)**, which include the recruitment of additional beneficial microbes into their microbiota **(C, D)**. Beneficial microbiota assembled by host organisms suppress fungal pathogen invasion through a diversity of mechanisms, including the secretion of antimicrobial molecules (purple and pink arrowheads) by some of the microbiota members **(E, F)**. Fungal invaders, however, evolved to counteract the suppressive activities of host microbiota using secreted effector proteins (white ‘Pac-man’ symbols) that modulate host microbiota compositions, for instance through the direct suppression of antagonistic microbiota members **(G, H)**, to facilitate host colonization and establish disease **(I)**.

Plants actively recruit mutualists and beneficial endophytes into their microbiota using exudates that promote the accommodation of these microbes (Bais *et al*. 2006, Rudrappa *et al*. 2008, Mendes *et al*. 2011, Berendsen *et al*. 2012, van der Heijden *et al*. 2015, Huang *et al*. 2019, Koprivova *et al*. 2019). Such beneficial microbes can colonize aboveground as well as belowground plant organs and contribute to plant health, for instance by promoting nutrient acquisition, enhancing abiotic stress tolerance or suppressing pathogen attack (Dimkpa *et al*. 2009, Innerebner *et al*. 2011, Vorholt 2012, Almario *et al*. 2017, Durán *et al*. 2018, Fitzpatrick *et al*. 2018, Lu *et al*. 2018, Stringlis *et al*. 2018, Carrión *et al*. 2019, Sarkar *et al*. 2019, Chen *et al*. 2020, Pfeilmeier *et al*. 2021, Yu *et al*. 2021). Its intimate association with the host, as well as the fundamental functions it performs for the plant, makes the microbiota an integral component of the plant when considered as a ‘holobiont’. In other words, plants are ‘meta-organisms’ composed of the plant itself plus the associated microbiota. Considering the positive impact of plant-associated microbial communities on plant health, basically as an extension of the endogenous immune system of the plant, it is conceivable that pathogens evolved to not only target the endogenous immune system of their plant hosts but also target the host-associated microbiota in order to mediate disease establishment (Snelders *et al*. 2018). Accordingly, we previously hypothesized that fungal plant pathogens may use effector proteins to manipulate the microbiota of their plant hosts in order to facilitate disease development (Snelders *et al*. 2018) (Fig. 1). Recent evidence supports this hypothesis (Snelders *et al*. 2020, Snelders *et al*. 2021). In this review, we examine the role of fungal effector proteins in the manipulation of microbial interactions. We address how such effectors may aid fungal symbionts of plants and discuss when and where these effectors may have evolved. We then explore further deployment of microbiota-manipulating effector proteins by fungi in other ecological contexts. Finally, we argue that fungi may exploit effector proteins for microbiota manipulation to support colonization of any holobiont.

## FUNGAL EFFECTOR-MEDIATED MANIPULATION OF PLANT HOST MICROBIOTA

### The soil-borne pathogen *Verticillium dahliae*

Although beneficial plant-associated microbes are found on all plant organs, root-associated microbes are most intensively studied with respect to their contribution to plant health. The microbial communities in the soil represent an enormous pool of largely uncharacterized biological diversity, and although soil is considered to be microbe rich, microbial proliferation is limited by the relatively poor nutrient availability in this biome (Demoling *et al*. 2007). Plants release a significant amount of their photosynthetically fixed carbon into the rhizosphere, the narrow zone of soil in close proximity to their roots. These nutrient-rich root exudates attract microbes, which leads to an increased microbial density in the rhizosphere when compared with the bulk soil, which makes it a competitive environment. The attraction of microbes through the secretion of root exudates, known as the ‘rhizosphere effect’, illustrates the intimate relationship between plants and their root microbiota (Berendsen *et al*. 2012). Importantly, plants define the composition of their rhizosphere and root endosphere microbiota using exudates and recruit beneficial microbial communities to suppress pathogen invasion and alleviate abiotic stress (Rudrappa *et al*. 2008, Mendes *et al*. 2011, Berendsen *et al*. 2018, Kwak *et al*. 2018, Carrión *et al*. 2019, Harbort *et al*. 2020).

Soil-borne pathogens display a diversity of lifestyles and exploit various infection strategies, but for most of them initial host colonization is established via the roots. Consequently, to cause disease, soil-borne plant pathogens first need to traverse the hostile host root microbiota. Considering the pivotal role of root-associated microbes in restricting pathogen invasion, we hypothesized that soil-borne fungal plant pathogens dedicate part of their effector repertoire to host microbiota manipulation (Snelders *et al*. 2018). Several metagenome analyses have revealed an impact of colonization by soil-borne fungal pathogens on root microbiome compositions (Mendes *et al*. 2011, Chapelle *et al*. 2016, Carrión *et al*. 2019, Gao *et al*. 2021). However, most of these community structure alterations have been interpreted in the light of root exudate-mediated recruitment of beneficial microbes that contribute to disease suppression, and the hypothesis that pathogens may simultaneously manipulate these communities is generally overlooked.

**Figure 2.**
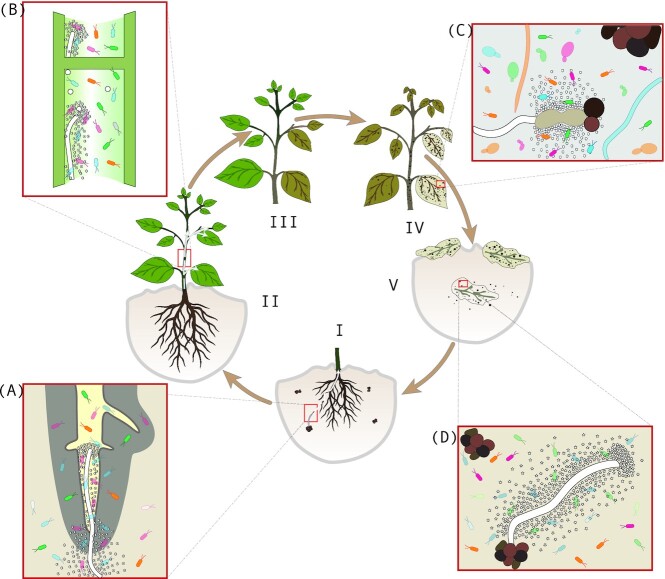
Life stage-specific effector-mediated microbiota manipulation by the soil-borne broad host-range fungal plant pathogen *V. dahliae*. The fungus survives in the soil through multicellular melanized resting structures called microsclerotia. Germination of microsclerotia is stimulated by plant root exudates, after which the emerging hyphae grow through the rhizosphere to penetrate plant roots **(I)**. Next, the fungus crosses the root cortex and endodermis to enter the xylem vessels where sporulation occurs **(II)**. The conidiospores are transported with the sap stream in the xylem to distal plant tissues. Once conidiospores get trapped, germination occurs, after which the fungus penetrates into new xylem vessels where sporulation reoccurs. This systemic colonization is accompanied by typical *Verticillium* wilt symptoms, including chlorosis, necrosis and wilting **(III)**. Once the tissue starts to senesce, *V. dahliae* emerges from the vasculature to colonize decaying host tissues where new microsclerotia are produced **(IV)**. These microsclerotia are released into the soil upon littering and tissue decomposition **(V)**. *Verticillium dahliae* secretes a plethora of effector proteins to promote host colonization, several of which target the diversity of microbiota that it encounters during its life cycle. VdAve1 is a ubiquitously expressed antibacterial effector that promotes colonization of the soil as well as the roots **(A)** and xylem vessels **(B)** of its hosts through selective suppression of antagonistic bacteria, including *Sphingomonas* species. **(C)** VdAMP3 is an antifungal effector protein that is specifically expressed to ward off fungal niche competitors, comprising yeasts and filamentous fungi, during microsclerotia formation in decaying phyllosphere tissues. **(D)** Finally, VdAMP2 is an antibacterial effector that is exclusively expressed in the soil where it complements the activity spectrum of VdAve1 to mediate soil survival.

We recently provided the first evidence that beneficial plant-associated microbial communities are targeted by fungal pathogens through the use of effector proteins. The soil-borne, xylem-invading plant pathogenic fungus *V. dahliae* secretes several effector proteins that display antimicrobial activity *in planta* to suppress microbial antagonists (Fig. 2) (Snelders *et al*. 2020, 2021). More specifically, particular strains of the fungus secrete the virulence effector VdAve1 (de Jonge *et al*. 2012), as a ubiquitously expressed antibacterial protein that impacts microbiota compositions of the roots and xylem of the host plants tomato and cotton (Snelders *et al*. 2020) (Fig. 2A and B). Intriguingly, secretion of this effector suppresses the proliferation of bacteria of the Sphingomonadales order that were shown to display antagonistic activity toward *V. dahliae* growth. Thus, secretion of VdAve1 promotes disease development by the fungus through selective targeting of microbial antagonists in the host plant microbiota. Interestingly, *V. dahliae* secretes not only VdAve1 during host colonization but also in soil where the fungus can thrive as a saprotroph in absence of a suitable host (Snelders *et al*. 2020).

Sequence similarity searches in the genomes of a diverse collection of *V. dahliae* strains revealed a multiallelic *VdAve1*-like (*VdAve1L*) gene. An allelic effector variant encoded by one of these genes, named VdAve1L2, shares substantial sequence similarity with VdAve1 and likewise exerts antibacterial activity (Snelders *et al*., unpublished). However, VdAve1L2 seems to have functionally diverged from VdAve1, as the VdAve1L2 effector displays a distinct activity against antagonistic root-associated Actinobacteria. Remarkably, in contrast to VdAve1, VdAve1L2 is strictly expressed during plant colonization and not during soil-dwelling stages (Snelders *et al*., unpublished).

Whereas VdAve1 and VdAve1L2 are lineage-specific effectors that occur only in particular strains of the *V. dahliae* population, one can argue that if manipulation of the host microbiota is fundamental to host colonization strategies, antimicrobial effector proteins should be found in the core effector repertoire of the species as well. When probing the *V. dahliae* effector repertoire for proteins with putative structural homology to known antimicrobial proteins (AMPs), ten additional effector candidates were identified that are potentially involved in microbiome manipulation (Snelders *et al*. 2020). One of these effectors, named VdAMP3, is conserved in the *V. dahliae* population and is specifically expressed at the end of the disease cycle, when phyllosphere plant tissues have become necrotic and the fungus emerges from the xylem to produce melanized resting structures called microsclerotia that are released in the soil upon littering of the plant tissues followed by tissue decomposition (Snelders *et al*. 2021) (Fig. 2C). Intriguingly, in contrast to VdAve1 and VdAve1L2 that exert antibacterial activity, VdAMP3 exerts antifungal activity to ward off opportunistic fungal niche competitors that act in the decay of host tissues (Snelders *et al*. 2021) (Fig. 2C). Collectively, these findings demonstrate that *V. dahliae* exploits antimicrobial effector proteins that display differential, and highly selective, activity. Moreover, these effectors are secreted in a life stage-specific manner to manipulate bacteriota and mycobiota in different host tissues. Arguably, VdAve1, VdAve1L2 and VdAMP3 may only represent a small proportion of the *V. dahliae* effector complement that is intended for host microbiota manipulation. Eight of the effector proteins that were predicted to share structural homology with known AMPs (Snelders *et al*. 2020) have remained uncharacterized till now, but are likely to comprise additional effectors involved in microbiota manipulation. It is important to realize that these effectors were identified based on their structural homology to known antimicrobials. However, the secretome of *V. dahliae* is likely to contain antimicrobials with folds or domains that are not recognized based on current structural models. In support of this notion, VdAve1 and VdAve1L2 share no homology with previously described antimicrobial proteins; the main reason why they were not anticipated to display antimicrobial activity at the time of their discovery. Thus, plant pathogen effector catalogues are likely to comprise novel antimicrobial proteins.

### Plant pathogens with diverse colonization styles

Considering the strong microbial competition in bulk soil and in root microbiota, it can be anticipated that soil-borne pathogens other than *V. dahliae* will benefit from the exploitation of effector proteins that act in microbiota manipulation. However, evidence for this hypothesis is limited at present. Recently, a putative β-lactamase effector secreted by pathogenic *Fusarium oxysporum* was demonstrated to impact the soybean root microbiota and to promote growth of the pathogenic fungus in the presence of the proteobacterium *Burkholderia ambifaria* in *in vitro* assays (Chang *et al*. 2021). However, the relevance for soybean colonization was not demonstrated in this study. Further evidence for the use of effector proteins by soil-borne fungal pathogens to manipulate intermicrobial interactions is exclusively based on transcriptional analyses. For instance, transcriptome analyses performed upon confrontation of the cereal fungal pathogen *Bipolaris sorokiniana* with the beneficial root endophyte *Serendipita vermifera* in soil and *in planta* revealed that the fungal pathogen expresses multiple genes encoding potential antimicrobial effectors, including putative chitinases (Sarkar *et al*. 2019). However, a role in its own growth and development, such as the facilitation of hyphal branching, cannot be excluded at present.

While the phyllosphere microbiota has been characterized less well, it is becoming increasingly clear that plants also assemble phyllosphere microbiota that benefit plant health, for instance through disease suppression (Innerebner *et al*. 2011, Vorholt 2012, Ritpitakphong *et al*. 2016, Berg and Koskella 2018, Chen *et al*. 2018, Pfeilmeier *et al*. 2021). Irrespective of their lifestyles, disease establishment by fungal pathogens in the phyllosphere of compatible host plants is typically initiated by spores that land on these tissues. These spores germinate and the emerging hyphae start to colonize the exterior of their hosts, the so-called episphere, the extent of which depends on the fungal colonization style. Next, penetration of the host tissue occurs, either directly or via natural openings such as stomata, after which host tissues are colonized.

Arguably, antimicrobial effectors can contribute both during and right after spore germination as they could support initial niche colonization in the already established phyllosphere microbial communities through the suppression of epiphytes in the immediate environment. Recently, the obligate biotrophic powdery mildew fungus *Golovinomyces orontii* was shown to outcompete resident leaf-associated fungi during colonization of *Arabidopsis thaliana* (Durán *et al*. 2021), which could point toward the exploitation of antifungal effector proteins by the pathogen during colonization of the episphere. This is similarly true for *Zymoseptoria tritici*, a foliar pathogen and causal agent of *Septoria tritici* leaf blotch of wheat that was shown to secrete the Zt6 effector during spore germination, which was shown to possess phytotoxic and antimicrobial ribonuclease activity (Kettles *et al*. 2018). Hence, Zt6 was speculated to clear the immediate surroundings of the germinating spore from microbial competitors to safeguard initial leaf colonization. However, targeted deletion of *Zt6* did not affect *Z. tritici* disease establishment, and currently the role of Zt6 in microbial inhibition *in planta* remains unclear.

Following epiphytic colonization of host tissues in the phyllosphere, plant infection by many fungal pathogens continues in the apoplast where, depending on the lifestyle, pathogens adopt different strategies to acquire nutrients from their hosts. While biotrophs only obtain nutrients from living plant tissue, necrotrophs actively kill host cells for nutrient acquisition (Glazebrook 2005). However, most pathogens can be placed somewhere in the continuum between these lifestyles and are classified as hemibiotrophs that initially establish a biotrophic interaction with their hosts that is succeeded at some point in time, for some sooner and for others later, by a necrotrophic phase (Spanu 2012). Importantly, extensive colonization of the apoplast is predominantly restricted to specialized microbes, including pathogens, with the ability to subvert host immunity using effector molecules (Rocafort *et al*. 2020). Consequently, the microbial densities encountered by phyllosphere-colonizing plant pathogens in the apoplast are generally much lower than in the episphere (Hunter *et al*. 2010, Chen *et al*. 2020), and the potential importance of antimicrobial effectors for the suppression of niche competitors in the apoplast could be rather limited. Nevertheless, mass spectrometry analyses on the apoplastic wash fluid obtained from tomato plants infected by the biotrophic fungal pathogen *Cladosporium fulvum* revealed that 10% of the identified *in planta*-secreted effector proteins share predicted structural similarity with antimicrobial proteins (Mesarich *et al*. 2018). Although effectors are typically small and rich in cysteines, and therefore likely to adopt tight toxin and defensin-like folds even without displaying such activity, the observation as made for *C. fulvum* may point toward the potential involvement of pathogen effector proteins in microbial competition in the leaf apoplast. Similarly, transcriptome analyses combined with protein structure predictions using AlphaFold2 revealed that the biotrophic apple scab fungus *Venturia inaequalis* expresses various effector proteins with predicted structural similarity to antimicrobial proteins during host colonization (Rocafort *et al*. 2022).

During necrotrophic infection stages, fungal pathogens actively induce plant tissue necrosis, which is accompanied by the dissipation of host immune responses, and make the plant tissue an attractive niche for opportunistic parasites. Hence, for pathogenic microbes, microbial competition is likely to increase during the transition from biotrophy to necrotrophy. Consequently, leaf pathogens with necrotrophic life stages may be anticipated to exploit antimicrobial effector proteins to protect their niche from the wealth of potential competitors that can emerge once host immune responses fade and plant tissue collapses. Although experimental evidence for this hypothesis is presently lacking, findings on the role of the *V. dahliae* effector VdAMP3 to safeguard microsclerotia formation in decaying host phyllosphere tissues underpin the relevance of the exploitation of antimicrobial effectors to ward off opportunistic competitors in necrotic host tissues (Snelders *et al*. 2021).

### Direct versus indirect pathogen effector-mediated microbiome manipulation

It needs to be noted that the impact of microbiota-manipulating effectors will generally be largely restricted to microbes that reside in close proximity to the invading fungus. Since plants and their associated microbes function as meta-organisms in which plant immunity and physiology are intimately linked with microbiota contributions, fungal effectors that target host physiology may inevitably also impact the host microbiota in an indirect manner. Accordingly, fungal pathogens may abuse this intertwined relationship between plants and their microbiota in an indirect or more systemic manner using effector proteins that influence endogenous plant processes. Interestingly, a recent characterization of wheat microbiomes revealed that *Z. tritici* induces local and systemic changes in leaf microbiota compositions during wheat colonization that are speculated to be the outcome of effector-mediated immune suppression and that are accompanied by increased susceptibility of leaf tissues toward pathogen infection (Seybold *et al*. 2020). Hence, fungal pathogens may manipulate microbiota compositions in distal plant tissues using effector proteins to facilitate subsequent colonization of these tissues, a strategy that may benefit fungal plant pathogens with diverse lifestyles.

### Effector-mediated microbiome manipulation by nonpathogenic fungal symbionts

Like their pathogenic relatives, endophytic and mutualistic fungi also secrete effector proteins to establish symbiotic relationships with their plant hosts (Kloppholz *et al*. 2011, Voss *et al*. 2018, Sarkar *et al*. 2019, Nostadt *et al*. 2020, Plett *et al*. 2020, Zeng *et al*. 2020). These symbionts encounter the same microbiota as fungal pathogens, and may therefore also secrete effector proteins to manipulate host microbiota compositions. A fundamental difference when compared with fungal pathogens, however, is that while pathogens aim to counteract the recruitment of beneficial microbes by their host (Fig. 1), proliferation of endophytes and mutualists is (in some cases) actively promoted by host plants to facilitate their accommodation in the microbiome (Akiyama *et al*. 2005, Besserer *et al*. 2006, Lombardi *et al*. 2018, Rich *et al*. 2021). Therefore, microbiota-targeting effector proteins secreted by nonpathogenic plant-associated fungi may act in concert with plant-derived molecules to establish themselves in host microbiota. This hypothesis is supported by the fact that several nonpathogenic fungal colonizers of plants have been demonstrated to express (putative) antimicrobial effector proteins during plant colonization to control other microbes (Li *et al*. 2004, Chen *et al*. 2009, Ambrose and Belanger 2012, Romao-Dumaresq *et al*. 2012, Guzman-Guzman *et al*. 2017, Eitzen *et al*. 2021). For example, the epiphytic yeast *Moesziomyces bullatus* ex *Albugo* was shown to antagonize infection by the oomycetal white rust pathogen *Albugo laibachii* on *Arabidopsis thaliana* through the secretion of a GH25 hydrolase with predicted lysozyme activity (Eitzen *et al*. 2021).

### Beneficial hyphosphere interactions in the plant holobiont context

Even though studies of microbial interactions in plant holobionts have predominantly addressed antagonism and competition, plant-associated fungi also engage in beneficial interactions with microbial coinhabitants (Partida-Martinez and Hertweck 2005, Arendt *et al*. 2016). Fungal hyphae and their immediate surroundings, the so-called ‘hyphosphere’, form microhabitats that are colonized by specialized microbial communities (Warmink *et al*. 2009, Stopnisek *et al*. 2016, Ghodsalavi *et al*. 2017, Schulz-Bohm *et al*. 2017, Deveau *et al*. 2018). Of all plant-associated fungi, microbial communities surrounding mycelial networks formed by arbuscular mycorrhizal fungi have been most well studied (Scheublin *et al*. 2010, Zhou *et al*. 2020, Emmett *et al*. 2021). Intriguingly, analogous to the role of plant root exudates in the assembly of root microbiota, multiple *in vitro* experiments provided evidence that (carbon-rich) fungal exudates stimulate the growth of particular bacteria and impact microbial community structures (Filion *et al*. 1999, Toljander *et al*. 2007, Warmink *et al*. 2009). Importantly, bacterial symbionts can fulfill beneficial activities for fungi through, for instance, facilitating the establishment of symbioses between arbuscular mycorrhizal fungi and plants, as well as the protection of fungi from antifungal compounds (Frey-Klett *et al*. 2007, Nazir *et al*. 2014). At the same time, bacteria derive benefits from the fungal association. It was recently demonstrated that bacterial lipopeptides induce the production of specialized fungal survival structures, chlamydospores, that act as reservoirs for non-endosymbiotic bacterial taxa that enter and propagate in these chlamydospores, resulting in higher fitness of these bacteria when challenged with abiotic stresses (Venkatesh *et al*. 2022).

Although evidence for intimate hyphosphere interactions between bacteria and fungi in the context of the plant holobiont is presently mostly limited to arbuscular mycorrhizal fungi and their ectosymbionts, fungal plant pathogens can similarly be anticipated to establish associations with microbiota coinhabitants. Fungal hyphae can act as vectors that facilitate migration of bacteria (Kohlmeier *et al*. 2005, Furuno *et al*. 2012, Nazir *et al*. 2012, Simon *et al*. 2015). Hence, fungal plant pathogens might recruit cooperative ectosymbiotic bacteria to migrate along their hyphae to support the colonization of plants. Bacterial symbionts could, for instance, aid in the suppression of antagonists or confer protection against antimicrobials secreted by plant hosts or their associated microbes. Alternatively, bacterial symbionts might even contribute to direct host manipulation. The fungal pathogen *Rhizopus microsporus*, causal agent of rice seedling blight, carries endosymbiotic *Mycetohabitans rhizoxina* bacteria that synthesize the phytotoxic metabolite rhizoxin that acts as a crucial virulence factor for rice colonization by the fungus (Partida-Martinez and Hertweck 2005, Lackner *et al*. 2011). In soil environments, *R. microsporus* is subject to predatory amoeba such as *Dictyostelium discoideum*, which feeds by engulfing microbial cells. To evade predation, *R. microsporus* relies on molecules secreted by its endosymbiont bacterium *Ralstonia pickettii*, which interfere with phagocytosis by *D. discoidenum* (Itabangi *et al*. 2022). Interestingly, this anti-phagocyte activity also protects *R. microsporus* from clearance by human macrophages during opportunistic infections, and *R. microscopus* virulence is greatly reduced in absence of the endosymbiont (Itabangi *et al*. 2022). Hence, the recruitment of microbial symbionts can extend the virulence potential of fungal pathogens and, possibly, effector proteins could play a role in such a recruitment. Although the initial attraction of bacteria to the hyphosphere might in part be based on exudates released by the fungus, effector proteins may aid in shaping the attracted community and could facilitate the accommodation of specific microbes. For instance, the secretion of antimicrobial effector proteins could enrich for beneficial symbionts that are tolerant to these proteins and therefore benefit from a competitive advantage over other microbes. Alternatively, effector proteins may establish physical interactions between plant pathogens and their symbionts. The importance of maintaining beneficial hyphosphere microbiota by fungal plant pathogens was recently underscored by the discovery that the endophytic rhizobacterium *Rahnella aquatilis* can hijack hyphae of *F. oxysporum* to efficiently colonize tomato roots where the bacteria subsequently antagonize disease establishment by the fungus (Palmieri *et al*. 2020).

### Effector-mediated microbiome manipulation during saprotrophism

Like free-living saprotrophs that thrive on decaying organic matter in the soil, many plant-associated fungi also undergo saprotrophic life stages outside their hosts in niches that are generally characterized by rich microbiota. Consequently, plant-associated fungi also secrete microbiota-manipulating effectors to establish themselves in such niches. The role of antibacterial effector proteins for niche colonization during saprotrophic stages in soil is underscored by the ubiquitously expressed *V. dahliae* effector VdAve1 that promotes soil colonization (Snelders *et al*. 2020) and the identification of additional effector proteins that act solely during these life stages. The *V. dahliae* effector VdAMP2, identified through its predicted structural homology with known AMPs, displays selective antibacterial activity and is exclusively expressed by *V. dahliae* during soil colonization (Snelders *et al*. 2020) (Fig. 2D). Colonization assays demonstrated that *VdAMP2* deletion mutants were less able to colonize soil than wild-type *V. dahliae*, an effect that was not observed in sterilized soil, and consequently it was concluded that besides VdAve1, also VdAMP2 is required for optimal competitiveness in this environment. Notably, VdAve1 and VdAMP2 display divergent activity spectra, suggesting that their activities complement each other for optimal soil colonization by *V. dahliae*. Thus, plant-associated fungi also exploit effector proteins outside their hosts in a life stage-specific manner to enhance their competitiveness in microbial communities. VdAve1 and VdAMP2 likely only represent two examples of a multitude of effector proteins that are secreted in this environment by a diversity of plant-associated fungi. This is supported by the existence of effector protein families that are conserved between facultative host-associated and strictly free-living fungi (Brown 2011, Kombrink and Thomma 2013, Kettles *et al*. 2018, Seidl and van den Ackerveken 2019), which hints toward exploitation of effectors with functions outside the plant holobiont, such as microbiota manipulation during saprotrophism. Accordingly, several potent antimicrobial effector proteins have been identified in saprotrophic fungi including *Aspergillus* and *Penicillium* species (Wnendt *et al*. 1994, Seibold *et al*. 2011, Contreras *et al*. 2018, Kombrink *et al*. 2019). In fact, based on such findings it may be argued that many of these effectors actually have their evolutionary origin outside the plant host (Snelders *et al*. 2021).

## THE EVOLUTION OF MICROBIOTA-MANIPULATING EFFECTORS

### Environmental niches and lifestyles of ancient fungi

The occurrence of fungal effector molecules with roles in microbiota manipulation both within and outside plant hosts, and the occurrence of fungal effector molecules with roles in microbiota manipulation in symbionts as well as free-living fungi, raises the question of their evolutionary origin. As heterotrophs, fungi rely on other organisms for nutrition and energy. Most fungi live as symbionts or saprotrophs that feed on plant-derived substrates, such as cellulose and hemicellulose. Recent phylogenomic analyses estimated that the earliest fungi emerged in the Precambrian period, possibly over 1000 million years ago, from phagotrophic protists that thrived in freshwater environments that also harbored a myriad of prokaryotes, including cyanobacteria and phototrophic eukaryotes, the ancestors to all green plants (Parfrey *et al*. 2011, de Vries and Archibald 2017, Ponce-Toledo *et al*. 2017, Loron *et al*. 2019, Berbee *et al*. 2020). The evolution of these fungal osmotrophs that fed on dissolved organic compounds likely depended on the acquisition of cellulase-encoding genes that enabled the earliest fungi to hydrolyze cell walls of cyanobacteria and streptophyte algae, the group of freshwater algae from which land plants evolved, to liberate essential nutrients (Berbee *et al*. 2017, Richards *et al*. 2017). Indirect evidence for this ancestral nutritional dependence is provided by the fact that a variation in extracellular polysaccharides secreted by cyanobacteria contributes to defense against fungal attack and has been hypothesized to have served as a barrier to digestion by early fungi (Agha *et al*. 2018, Berbee *et al*. 2020).

### Ancient biota as drivers for the emergence of microbiota-manipulating effectors in ancestors of extant fungi

**Figure 3.**
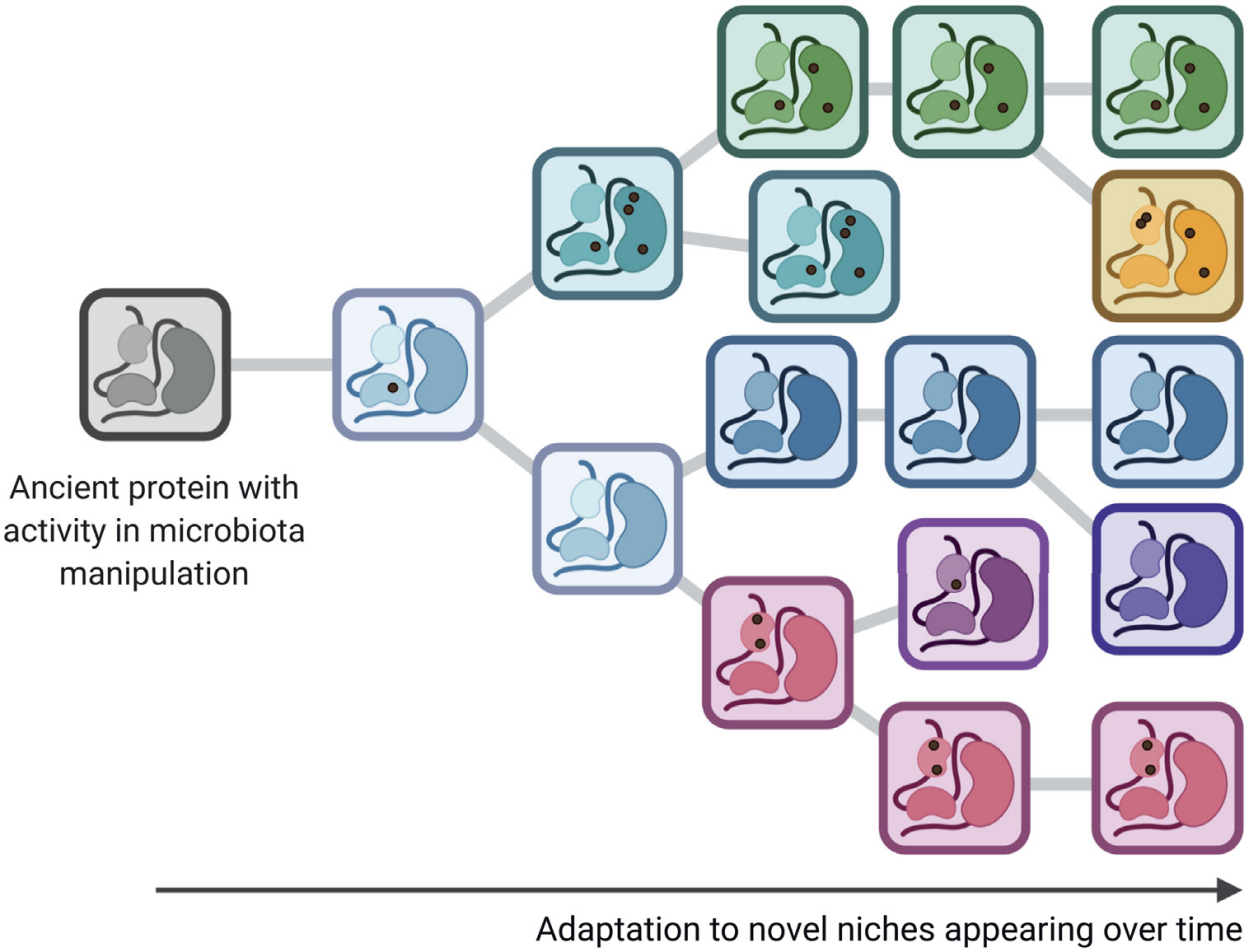
Evolution of microbiota-manipulating effectors from proteins of ancient fungi. The first fungi emerged long before more complex multicellular life forms, such as plants and animals, evolved. They colonized early microbe-rich aquatic and terrestrial ecosystems, presumably in the shape of microbial mats (or biofilms) that may have provided shelter from biotic and abiotic stress. The combination of microbial cooperation required for the establishment in dense microbial communities and the resulting competition for space and nutrients likely drove the evolution of microbe-manipulating effector-like proteins in the ancestors of extant fungi. Such effectors may have been maintained and their functions co-opted through minor and major (structural) changes (illustrated by black dots) as fungi colonized and adapted to novel niches (different colors) over time.

Symbioses of early terrestrial fungi undoubtedly were not restricted to cellulose-synthesizing microbes alone, as the terrestrial environments colonized by these fungi were also home to prokaryotes. Additionally, considering that extant algae also assemble and shape their own bacteriota (Knack *et al*. 2015, Durán *et al*. 2022) it seems inevitable that the earliest fungi interacted with algae-associated microbes too. These interactions are believed to have taken place in microbially complex niches such as (multispecies) microbial biofilms, crusts or mats (Noffke *et al*. 2006, Tomescu *et al*. 2009, Brasier *et al*. 2010), each with their own structural and spatial organization and corresponding evolutionary dynamics (Ereshefsky and Pedroso 2015, Clarke 2016, Kayser *et al*. 2018). Extant biofilms are known to provide benefits to their inhabitants, for instance by offering protection to a multitude of stress agents including ultraviolet radiation, extreme temperatures, drought and antimicrobials (Yin *et al*. 2019). Assuming that ancient biofilms offered similar advantages, it may be likely that early fungi already evolved tools, including secreted effector(-like) proteins, to actively establish beneficial microbial symbioses. While microbes often benefit from biofilm formation, biofilms are also characterized by extensive microbial competition (Nadell *et al*. 2016). Hence, the interaction of early fungi with microbial coinhabitants in biofilms likely involved the secretion of antimicrobials to cope with competition and to facilitate niche establishment. Indeed, the existence of fungal antimicrobial proteins of which the origin dates back to before the first fungal lineages diverged (Snelders *et al*. 2021), indicates that early fungi already exploited antimicrobials (Shafee *et al*. 2016). Considering that this origin preceded the evolution of land plants, it seems likely that a substantial number of fungal effectors involved in host microbiota manipulation may have evolved from ancient proteins of fungal ancestors that served in microbial symbioses in early aquatic and terrestrial ecosystems prior to the emergence of land plants and the evolution of plant pathogenicity (Fig. 3). For example, the *V. dahliae* protein VdAMP3 belongs to the cysteine-stabilized αβ (CSαβ) defensins, a widespread and well-characterized family of antimicrobial proteins that originates from the last common ancestor of animals, plants and fungi, many of which still produce these proteins today. Thus, VdAMP3 has an ancient origin that predates the evolution of land plants, was likely implicated in intermicrobial competition, and has been co-opted by *V. dahliae* as an effector for the manipulation of the host mycobiota (Snelders *et al*. 2021). Obviously, plant pathogens may also have evolved microbiome manipulating effectors more recently, for instance as part of the molecular coevolution with vascular plants and the associated microbiota. Undeniably, the antibacterial *V. dahliae* effector VdAve1 is one such protein, as the gene encoding this virulence factor was acquired by horizontal transfer from a higher plant where homologs ubiquitously occur and are generally annotated as plant natriuretic peptides or as expansin-like proteins (de Jonge *et al*. 2012).

### Microbial coevolution in extant holobionts favors emergence of effector proteins that target microbiota core taxa

The lifestyles and host ranges of plant pathogens are likely to determine the degree of coevolution with host-associated microbes. Many fungal plant pathogens are facultative pathogens that spend part of their life cycle in association with a host, while spending the remainder of that life cycle in other niches. Environmental biomes like soils are generally characterized by more diverse microbiota than plants that are enriched in a subset of taxa attracted from the environment. Hence, facultative pathogens are exposed to a larger breadth of microbes than obligate biotrophic pathogens that cannot survive in absence of their host. While environmental factors like the climate and the soil (micro)biome largely impact plant microbiota compositions, different plant species and genotypes grown in the same environment still assemble distinct microbiota (Bulgarelli *et al*. 2015, Tkacz *et al*. 2020, Oyserman *et al*. 2021). As a consequence, broad host range pathogens potentially evolved molecular tools to interact with a larger diversity of microbes than specialist pathogens that exclusively cause disease on a single host or even host genotype. With respect to microbial competition, it seems favorable for facultative and/or broad host range pathogens to evolve antimicrobial effectors with broad spectrum activities that can impact a large diversity of microbes in many different niches. Remarkably, however, the microbiota-manipulating *V. dahliae* effectors characterized thus far display highly selective activities, and are expressed in a life stage-specific manner, making the diversity of microbes directly inhibited by the individual effectors limited (Snelders *et al*. 2020, 2021). Although it cannot be excluded that constitutively expressed pathogen effectors with broad spectrum antimicrobial activities exist, our findings indicate that at least some pathogen effectors evolved specialized antimicrobial activities tailored to target specific microbes in distinct niches. The observation that the microbes that are targeted by these effectors appear to be antagonists of *V. dahliae* growth suggests that they are the result of a long coevolutionary trajectory.

The *in planta* secreted microbiota-manipulating *V. dahliae* effectors VdAve1 and VdAve1L2 display selective antibacterial activities and were demonstrated to impact Sphingomonadales and Actinobacteria, respectively (Snelders *et al*. 2020) (Snelders *et al*., unpublished). Both bacterial orders are found on virtually any plant in any environment and represent core taxa of plant microbiota and, importantly, are known antagonizers of plant pathogens (Müller *et al*. 2016, Berendsen *et al*. 2018, Lee *et al*. 2021, Vogel *et al*. 2021). Based on these findings one could thus speculate that the continued coevolution of fungal broad host-range pathogens with plant holobionts predominantly favors the evolution of effectors that, despite narrow activity spectra, function in nearly any holobiont. An obvious advantage of targeting core microbes, besides their ubiquity in plant microbiota, is the fact that those often function as keystone taxa that largely impact microbial community structures (Carlstrom *et al*. 2019). The targeting of these taxa may more easily lead to manipulation of healthy plant microbiota compositions, which makes these microbes interesting targets of fungal pathogens in the coevolution with plant-associated microbial communities.

### Effector functions are the result of continued coevolution within a given holobiont

While plant-associated microbes undoubtedly represent major drivers for the evolution of microbiota-manipulating effectors of fungal symbionts, host plants also impose selection pressure on these effector proteins. As is evident from the recognition of the *V. dahliae* effector VdAve1 by the immune receptor Ve1 (Kawchuk *et al*. 2001, Fradin *et al*. 2009), plants evolved to recognize microbiota-manipulating effectors, like they also recognize effectors that target host physiology. As a consequence, plant pathogens may mutate or lose their microbiome-manipulating effectors to evade recognition, leading to pathogen races with different suites of microbe-targeting effectors, as is the case for *V. dahliae* race 2 strains that are characterized by lack of the *VdAve1* gene (de Jonge *et al*. 2012). We previously suggested that pathogen effector proteins could be broadly classified into three groups: plant-targeting effectors, microbe-targeting effectors, and multifunctional effectors targeting plants and microbes (Snelders *et al*. 2018). Arguably, effectors from the latter group exhibiting phytotoxic and antimicrobial activity would represent exquisite tools for necrotrophs or hemibiotrophs to simultaneously induce host cell death and suppress microbial competitors. An example of such effector is the previously mentioned phytotoxic and antimicrobial ribonuclease effector Zt6 from the wheat pathogen *Z. tritici* (Kettles *et al*. 2018). However, the expression of such effectors during non-necrotrophic life stages or by pathogens with biotrophic lifestyles may hamper host colonization, as damage to host cells may betray attempted pathogen ingress. Transcriptional analyses revealed that the *V. dahliae* effector VdAMP2 is expressed during soil colonization, but not during host colonization (Snelders *et al*. 2020). Interestingly, when transiently expressed in *N. benthamiana* leaves VdAMP2 induces tissue necrosis, pointing toward phytotoxicity of the effector (Snelders and Thomma, unpublished data). *Verticillium dahliae* thus likely represses VdAMP2 expression *in planta* to limit host cell damage to not compromise colonization. These findings underline the idea that the activities of extant microbiota-manipulating effectors are not merely the result of coevolution with (host-associated) microbes. Rather, they are the result of coevolution with plants as holobionts once fungi started to establish more intimate relationships with terrestrial plants.

## FUNGAL EFFECTOR-MEDIATED MANIPULATION IN A WIDE DIVERSITY OF ‘HOLOBIONT’ CONTEXTS

**Figure 4.**
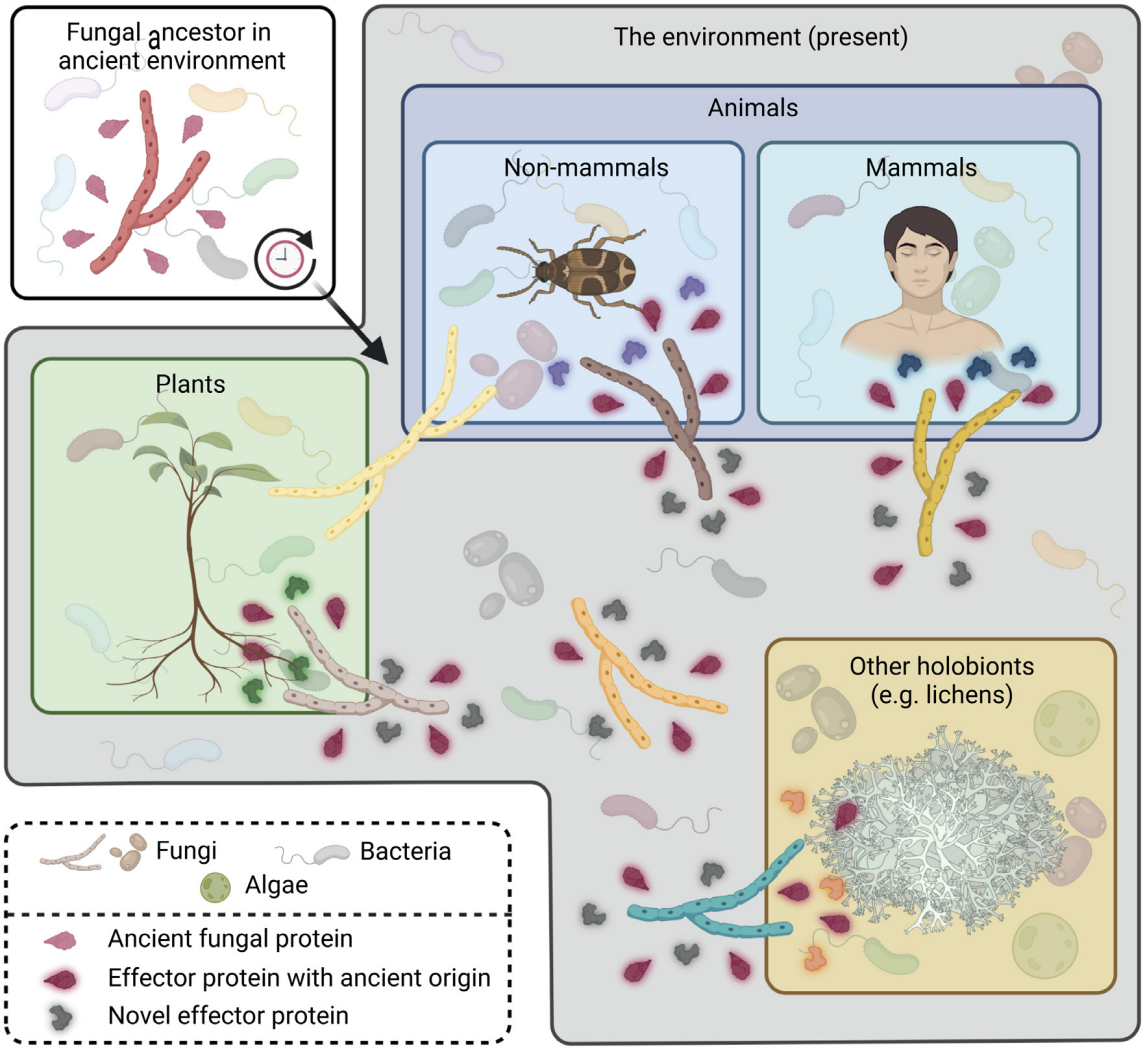
Fungi with diverse lifestyles exploit effector proteins for holobiont manipulation. Fungi are found in virtually any environment on Earth where they encounter a wealth of other microbes. Some fungi evolved symbiotic lifestyles and interact with multicellular hosts such as plants and animals that, together with their beneficial associated microbial communities, can be seen as holobionts. To promote the establishment of symbioses, fungi secrete effector proteins that influence such holobionts, for instance through the modulation of host physiological processes or immune responses. Additionally, fungi also exploit effector proteins, some with antimicrobial activities, to manipulate host microbiota compositions and stimulate holobiont colonization. A subset of these fungal effector proteins involved in microbiota manipulation evolved from ancient proteins of fungal ancestors that already interacted with other microbes long before fungi evolved as symbionts of plants, animals and other holobionts.

Building on the notion that microbiota manipulation has likely evolved in fungal ancestors that colonized early aquatic and terrestrial environments long before the emergence of vascular plants, microbiota-manipulating effectors may play roles in any contemporary context where fungi occur. This includes any type of symbiosis and, accordingly, any type of holobiont that fungi try to colonize. Extant fungal species colonize a diversity of microbe-rich niches in terrestrial habitats, where the exploitation of microbiota-manipulating effectors is expected to promote niche colonization (Fig. 4).

### Fungal pathogens of animals

Many fungal pathogens of mammals, including humans, are opportunistic pathogens that only cause disease in immunocompromised hosts that have contracted these fungi from the environment where they thrive as saprotrophs. Most of these pathogens display no host specificity and transmission from one individual to the other is rare, which accounts for very few reported cases of coevolution with their hosts (Thomma *et al*. 2011, Barber *et al*. 2021). Hence, the probability that novel effector proteins emerge as a consequence of coevolution with mammals seems low. This hypothesis is supported by the observation that, despite the fact that secretomes of fungal pathogens of mammals comprise a myriad of effector-like proteins (Lowe and Howlett 2012), the number of effector proteins that are reported to directly manipulate mammalian hosts is limited (Luberto *et al*. 2003, Moyes *et al*. 2016, Dasari *et al*. 2018, König *et al*. 2021). Considering that these fungi only incidentally cause disease, and predominantly thrive as saprotrophs, most effectors will be exploited during free-living life stages and may, for instance, play pivotal roles in interactions with microbial coinhabitants. During opportunistic infection of mammals, fungal pathogens also encounter a diversity of host-associated microbes, including antagonists. Hence, as a prerequisite to cause disease, fungal pathogens often need to successfully establish themselves in host microbiota. Considering the limited coevolution with the animal holobiont, during host colonization environmentally contracted fungal pathogens may rely on microbiota-manipulating effectors that (initially) evolved to contribute to their survival in the environment (Fig. 4). Importantly, symbiosis of opportunistic fungal pathogens with host-associated microbes is not restricted to commensals. For instance, the fungal pathogen *Aspergillus fumigatus* and the bacterial pathogen *Pseudomonas aeruginosa* frequently co-colonize the airways of immunocompromised patients with cystic fibrosis, where the mutual exploitation of secreted molecules, including proteins, to influence each other's growth, determines the disease process (Mowat *et al*. 2010, Moree *et al*. 2012, Briard *et al*. 2016, 2017, Margalit *et al*. 2020).

Besides environmentally contracted opportunistic pathogens, mammals can also suffer from opportunistic infections by fungal commensals that inhabit their own microbiota. Considering that such commensals are generally well controlled by host immune systems and only incidentally cause disease, these pathogens may not possess more sophisticated repertoires of effectors for manipulation of their hosts or the associated microbiota when compared with other commensals. Nevertheless, the commensal and opportunistic pathogen *Candida albicans* secretes a peptide toxin named Candidalysin that lyses epithelial cells and acts as a virulence factor for mucosal infection (Moyes *et al*. 2016). Additionally, the fungus simultaneously exploits the effector protein Pra1 (pH-regulated antigen 1) to sequester zinc from endothelial cells, which also contributes to virulence and host cell damage (Citiulo *et al*. 2012). *PRA1* homologs are conserved across higher and lower fungi, indicating that the ancestral gene of this effector arose in an ancient fungal lineage that predates the existence of animals (Fig. 3), and which potentially exploited this gene for sequestration of zinc from the environment. Hence, the discoveries of Candidalysin and Pra1 indicate that opportunistic pathogens also evolve effectors, in some cases from ancient proteins that previously served nonpathogenic lifestyles. Whether the exploitation of effector proteins by commensal opportunistic fungal pathogens is restricted to the interaction with their hosts, or also extends to the interaction with other host-associated microbes presently remains unknown. However, it is conceivable that they depend on microbiota-manipulating effectors too, to maintain themselves in the host microbiota (Fig. 1). Experimental evolution of *C. albicans* in the gastrointestinal tracts of antibiotic-treated mice was previously shown to induce mutations that perturb the ability to form hyphae, which renders the fungus avirulent (Tso *et al*. 2018). Importantly, although the hyphal-defective variants display an increased competitive fitness in antibiotic-treated mice over hyphae-forming strains, they fail to survive in the presence of an intact microbiota. Thus, the selective pressure exerted by the microbiota outweighs the selective pressure of the host, which underlines the necessity for molecular tools for opportunistic pathogens like *C. albicans* in the interaction with microbial coinhabitants.

While genuine fungal pathogens of mammals are rare, animals from other phyla are more frequently subject to transmissible fungal diseases. For instance, the fungal families Ophiocordycipitaceae and Ordycipitaceae comprise many notorious pathogens of insects. Comparative analyses of their genomes with those of ascomycete plant pathogens revealed that entomopathogenic fungi encode similar numbers of effector-like small cysteine-rich secreted proteins (Shang *et al*. 2016). While it is tempting to speculate that entomopathogenic fungi rely on effectors to promote disease, the characterization of their effector-like proteins is lagging behind when compared with those of fungal plant pathogens. Nevertheless, one of the best studied entomopathogenic fungi, *Beauveria bassiana*, was recently demonstrated to secrete a small cysteine-rich effector protein with antifungal and antioomycetal activity from its spores, presumably to inhibit filamentous niche competitors (Tong *et al*. 2020). Additionally, the fungus exploits two chitin-binding lysin motif (LysM) effectors, named Blys2 and Blys5, to promote virulence through the evasion of insect immune responses (Cen *et al*. 2017). LysM effector proteins are widely distributed in the fungal kingdom but have predominantly been studied in plant-pathogenic fungi where they act as suppressors of chitin-triggered plant immune responses (de Jonge *et al*. 2010, Sánchez-Vallet *et al*. 2015). Introduction of the virulence gene *MoSlp1*, encoding a LysM effector protein of the rice blast fungus *Magnaporthe oryzae*, in the genome of *B. bassiana* can compensate the virulence penalty that is introduced upon deletion of *Blys2* and *Blys5* (Cen *et al*. 2017). In light of this finding, it is relevant to note that many ascomycete entomopathogenic fungi, including *B. bassiana*, evolved from the same fungal lineages as (pathogenic) plant-associated fungi (Wang and Wang 2017), and that entomopathogenic fungi frequently also colonize plants as endophytes (Ownley *et al*. 2008, Behie *et al*. 2012). Importantly, nonpathogenic plant-associated fungi also rely on (LysM) effector proteins to establish symbioses with their hosts (Wawra *et al*. 2016, Nostadt *et al*. 2020, Zeng *et al*. 2020). Hence, it is plausible that effectors secreted by entomopathogenic fungi that modulate conserved processes, like Blys2 and Blys5, may promote different lifestyles in different holobiont contexts: a pathogenic lifestyle in insects and an endophytic lifestyle in plants.

To promote disease progression and host death, many entomopathogenic fungi secrete toxins during colonization of the insect hemocoel. While the vast majority of these molecules are secondary metabolites, they also compromise insecticidal effector proteins (Quesada-Moraga and Vey 2004). Intriguingly, *B. bassiana* secretes a secondary metabolite toxin named oosporein to down-regulate mosquito midgut immune responses, which induces dysbiosis in the gut microbiota and translocation of the opportunistic pathogenic bacterium *Serratia marcescens* into the hemocoel where it promotes mosquito death (Wei *et al*. 2017). Similarly, infection by *B. bassiana* also induces dysbiosis in red turpentine beetle gut microbiota, which accelerates its death. The ability to cause dysbiosis in their hosts is not restricted to insect pathogens. The pathogenic chytrid fungus *Batrachochytrium dendrobatidis*, responsible for severe declines of amphibians worldwide, was demonstrated to impact the skin bacteriota of frog species during infection (Jani and Briggs 2014, Jani *et al*. 2021). Similarly, abundance of the related fungal species *Batrachochytrium salamandrivorans* during infection of Salamanders was shown to correlate with skin bacteriota compositions, pointing toward pathogen-induced dysbiosis (Bates *et al*. 2019). Effector proteins secreted by fungal pathogens of insects or amphibians have not yet been demonstrated to modulate host microbiota. Nevertheless, the lifestyles of these microbes as genuine and transmissible pathogens, that involve extensive coevolution with animal microbiota, make effector-mediated microbiota manipulation an obvious infection strategy (Fig. 4), which is likely more important for these pathogens than for the causal agents of opportunistic diseases in animals.

### The mycobiont in lichen symbioses

In addition to saprotrophic fungi or those that associate with animals or vascular plants, ∼20 000 extant fungal species form stable, mutualistic symbioses with microalgae and/or cyanobacteria commonly referred to as lichens (Lücking *et al*. 2016). Lichens are ubiquitous, dominating ∼7% of the Earth's terrestrial surface (Asplund and Wardle 2017), and play an important role in extant biocrust ecosystems through nutrient (C and N) cycling, modification of soil properties and mineral weathering (Banfield *et al*. 1999, Belnap *et al*. 2016, Nelsen *et al*. 2020). Together, the fungal mycobiont and its algal/cyanobacterial partner (photobiont) form a 3D structure, called a thallus, where the fungal hyphae form a scaffold structure that embeds the photobiont, protecting it from desiccation, UV irradiation and other stresses (Solhaug *et al*. 2003, Hauck *et al*. 2008, Kranner *et al*. 2008, Nguyen *et al*. 2013). Since lichen-forming fungi are rarely observed in their free-living state in nature (Honegger 2009), it has long been assumed that the photobiont feeds the mycobiont with photosynthetically derived carbohydrates (Jahns 1988, Ahmadjian 1993, Grimm *et al*. 2021). This view has recently been challenged by the finding that the evolutionary adaptation to a lichen-forming lifestyle has not resulted in a consistent loss of mycobiont genes encoding carbohydrate-active enzymes, specifically plant cell wall degrading enzymes (Resl *et al*. 2021). This suggests that at least some lichen-forming fungi retained the ability to retrieve carbohydrates from sources other than their photobiont partner (Resl *et al*. 2021).

While often described as a one-on-one symbiosis, lichens do not only consist of a mycobiont and a photobiont partner but are, in fact, highly complex interkingdom communities including additional fungi, yeasts, bacteria and viruses (Aschenbrenner *et al*. 2016, Spribille *et al*. 2016, Fernández-Mendoza *et al*. 2017, Muggia and Grube 2018, Hawksworth and Grube 2020, Grimm *et al*. 2021). Reminiscent of plant-associated microbiota, lichen microbiota members contribute to nutrient cycling and acquisition, promote growth and enhance (a)biotic stress resistance (Grube *et al*. 2009, Cernava *et al*. 2015, 2017, Grube *et al*. 2015, Sigurbjörnsdóttir *et al*. 2015, Garg *et al*. 2016, Eymann *et al*. 2017, Almendras *et al*. 2018, Grimm *et al*. 2021). However, in addition to beneficial microbes, the microbial communities associated with lichens also comprise members with parasitic lifestyles (Lawrey and Diederich 2003). Lichens thrive in extreme environments, often under adverse climate conditions (Armstrong 2017). Therefore, they may act as a haven for microbes that occur in the same niche, as opportunistic invasion of lichen communities may help to escape from adverse environmental conditions. Accordingly, mycobionts may exploit effector proteins to attract beneficial microbes and fend off antagonists (Fig. 4). Similarly, fungal effectors proteins may facilitate lichen formation, growth and maintenance by directly manipulating their photobiont partners. Despite the fact that the evolutionary origin of lichens succeeded plant terrestrialization (Nelsen *et al*. 2020), it is conceivable that such effector proteins arose before plants colonized land in fungal ancestors that interacted with early plants, i.e. unicellular algae, and other microbes in aquatic environments (Fig. 3). It is tempting to speculate that effector proteins with ancient origins also facilitate other types of symbioses between extant fungi and algae as synthetic consortia of non-lichen-forming fungi and algae can establish long-term mutualistic relationships (Picard *et al*. 2013, Hom and Murray 2014, Simon *et al*. 2017, Du *et al*. 2019), suggesting an inherent ability of fungi to interact with algae. However, other fungal effectors may still have been obtained following the coevolution with algal symbionts in nature.

## CONCLUDING REMARKS

In this review, we addressed the diverse roles that secreted effector proteins play for fungi with various lifestyles. We argued that besides their well-established roles as modulators of physiological processes in multicellular hosts to support host colonization, fungal symbionts, including pathogens, secrete effector proteins to modulate host-associated microbiota compositions to their benefit (Fig. 1). Furthermore, we argued that effector-mediated microbiota manipulation may be relevant to the biology of any fungus in any ecological context, may be part of a general strategy to positively influence the environment, and is likely a trait that already evolved in the ancestors of extant fungi that occurred on Earth before land plants and mammals evolved (Fig. 4). The present-day contexts in which fungi may benefit from effector-mediated microbiota manipulation include a wide range of holobionts such as plants, insects and mammals, but also microbial holobionts such as lichens and fungal mycelial networks with their associated microbes. Although this review strongly focused on effector proteins, fungal manipulation of microbiota in holobiont contexts will likely not be determined by the activities of effector proteins alone, as fungi are well-known producers of bioactive ‘secondary metabolite’ molecules that include antibiotics such as penicillin (Scharf *et al*. 2014, Collemare *et al*. 2019). Furthermore, although less well established in this context, even small RNAs may be exploited to manipulate holobionts during fungal colonization (Weiberg *et al*. 2013, Wang *et al*. 2016). Finally, it is important to note that microbial communities in holobiont contexts may perhaps be dominated by bacterial and fungal taxa, but also contain other microscopic organisms such as oomycetes, protists, nematodes and arthropods. Given that among all these groups of organisms fungal feeding occurs, it is highly likely that fungi exploit molecules to target these organisms as well. In fact, the genome of the nematode-trapping fungus *Arthrobotrys flagrans* contains over 100 putative effector genes (Youssar *et al*. 2019), at least one of which contributes to *A. flagrans* virulence toward *Caenorhabditis elegans* (Wernet *et al*. 2021). The continued characterization of fungal effector repertoires, and the identification of further molecules involved in microbiota manipulation and their modes of action, may lead to the identification of novel bioactive molecules. Ultimately, this may result not only in an increased understanding of how microbial communities are assembled and shaped based on intermicrobial interactions but also in the development of novel antibiotics or food preservatives, and could contribute to the development of improved biocontrol strategies in crops and of improved application of probiotics in humans and animals.

## ACKNOWLEDGMENTS

The authors thank Fantin Mesny, Michael Seidl and David Cook for providing valuable feedback on an earlier version of the manuscript and Iliana Boshoven, owner of Agilecolor, for help with figure design.

## FUNDING

BPHJT acknowledges funding by the Alexander von Humboldt Foundation in the framework of an Alexander von Humboldt Professorship endowed by the German Federal Ministry of Education and Research and is furthermore supported by the Deutsche Forschungsgemeinschaft (DFG, German Research Foundation) under Germany's Excellence Strategy, EXC 2048/1, Project ID: 390686111.
